# Supplementary material for: Polyketide synthase gene clusters in the ericoid mycorrhizal fungus Oidiodendron maius: genome-wide prediction, expression profiling, and functional characterization of knockout mutants
Source: Front Plant Sci. 2026 Sep 16;17:1924430. doi: 10.3389/fpls.2026.1924430 (PMC13623903; doi:10.3389/fpls.2026.1924430)
Supplement: Supplementary file 1 [file SupplementaryFile1.zip › Supplementary Materials/Supplementary Folder 1.zip!/Oidma1_AssemblyScaffolds_Repeatmasked/knownclusterblast/cluster16/jgi.p_Oidma1_27678_mibig_hits.html]

| MIBiG Protein | Description | MIBiG Cluster | MiBiG Product | % ID | % Coverage | BLAST Score | E-value |
| --- | --- | --- | --- | --- | --- | --- | --- |
| AAC62818.1 | putative\_fatty\_acid\_synthase\_beta | BGC0000370\_c3 | HC-toxin\_biosynthetic\_gene\_cluster | 46 | 95.867768595 | 113 | 1.4e-25 |
| AGN71614.1 | fatty\_acid\_synthase\_beta\_subunit | BGC0000027 | Azaphilone\_biosynthetic\_gene\_cluster | 45 | 90.9090909091 | 98 | 6.2e-21 |
| AAC49199.1 | putative\_fatty\_acid\_synthase\_beta\_subunit | BGC0000152 | Sterigmatocystin\_biosynthetic\_gene\_cluster | 54 | 66.9421487603 | 97 | 1.4e-20 |
| ACH72900.1 | AflB | BGC0000011 | Aflatoxin/sterigmatocystin\_biosynthetic\_gene\_cluster | 50 | 76.8595041322 | 92 | 4.5e-19 |
| AAS90050.1 | HexB | BGC0000009 | Aflatoxin\_biosynthetic\_gene\_cluster | 47 | 79.3388429752 | 89 | 3.8e-18 |
| BAE71317.1 | fatty\_acid\_synthase\_beta\_subunit | BGC0000004 | Aflatoxin\_biosynthetic\_gene\_cluster | 46 | 91.7355371901 | 88 | 6.4e-18 |
| AAS90095.1 | Fas-1/HexB | BGC0000006 | Aflatoxin\_biosynthetic\_gene\_cluster | 46 | 91.7355371901 | 88 | 6.4e-18 |
| AAS90025.1 | HexB | BGC0000008 | Aflatoxin\_biosynthetic\_gene\_cluster | 46 | 91.7355371901 | 88 | 6.4e-18 |
| AAS90085.1 | HexB | BGC0000010 | Aflatoxin\_biosynthetic\_gene\_cluster | 46 | 91.7355371901 | 88 | 6.4e-18 |
| AAS90002.1 | HexB | BGC0000007 | Aflatoxin\_biosynthetic\_gene\_cluster | 46 | 78.5123966942 | 86 | 1.9e-17 |
| XP\_011392728.1 | fatty\_acid\_synthase\_FAS2 | BGC0001281 | Ustilagic\_acid\_biosynthetic\_gene\_cluster | 41 | 85.9504132231 | 80 | 1.3e-15 |
